# Large language models prompt engineering as a method for embodied cognitive linguistic representation: a case study of political metaphors in Trump’s discourse

**DOI:** 10.3389/fpsyg.2025.1591408

**Published:** 2025-06-25

**Authors:** Haohan Meng, Xiaoyu Li, Jinhua Sun

**Affiliations:** College of International Studies, National University of Defense Technology, Nanjing, China

**Keywords:** cognitive linguistics, large language models, metaphor identification, political discourse, embodied cognition, american studies

## Abstract

Embodied-Cognitive Linguistics inherits and further develops the core concepts of Cognitive Linguistics, maintaining a focus on embodied cognition and conceptual metaphors. It emphasizes that language is not merely a cognitive phenomenon but also a product of human social interactions and economic conditions. From this perspective, metaphors extend beyond their simple linguistic representation and become essential structures of human cognitive expression. Political metaphors, in particular, are instrumental in shaping public ideology and emotional engagement, a phenomenon clearly demonstrated in the political speeches of Donald Trump. With rapid advancements in large language models (LLMs) technology, traditional approaches to metaphor identification are undergoing significant transformation. By leveraging the advanced text parsing and generation capabilities of LLMs, new opportunities emerge for the automatic detection and nuanced analysis of political metaphors. This study employs a critical metaphor analysis (CMA) framework, integrated with a chain-of-thought-based prompt engineering (PE) technique, utilizing the ChatGPT-4.0 Python environment to identify and examine metaphors in Trump’s speeches. The results reveal that Trump strategically employs metaphors derived from diverse source domains—such as Movement and Direction, Illness and Health and Force—to resonate emotionally with his audience. Methodologically, while LLMs demonstrate considerable strengths in analyzing political discourse, challenges remain in areas such as semantic differentiation and expression. Future research will focus on optimizing these models, conducting comparative analyses with traditional methods, and exploring their applicability in cross-cultural contexts, with the goal of providing more precise and effective tools for both natural language processing (NLP) and political linguistics research.

## 1 Introduction

In the wake of the post-cognitivism revolution, embodied cognition has emerged as a central focus in cognitive psychology, asserting that cognition is fundamentally shaped by bodily experiences and actions. This perspective emphasizes the role of the body in forming conceptual frameworks, which are further informed by the body’s interactions with the environment (Gibbs, 2006, p.9). In alignment with this, metaphors, as Lakoff and Johnson argue, are not mere linguistic embellishments but essential to shaping our thoughts, actions, and worldviews (Lakoff and Johnson, 1980). They connect abstract concepts (the target domain) with familiar, concrete experiences (the source domain), facilitating cognitive coherence and conceptual evolution. In fact, the things that humans were initially most familiar with are the environment and space directly perceived by their bodies, “Our bodies and the interaction between our bodies and the world provide the most primitive concept for us to understand the world” (Ye, 2010). It is based on the prototype concept centered around the body that humans develop more abstract concepts and terms through metaphorical reasoning. As such, the role of metaphors extends to how we cognitively construct and interpret the world.

The physical and mental status of human beings cannot be separated from space and concrete materials, and cannot be separated from metaphors (Zhou and Luo, 2024). Metaphors, especially in the political realm, usually serve as a powerful tool to simplify and communicate abstract social ideas, anchoring them in shared experiences and resonating with audiences’ subconscious symbolic systems. This process not only aids in understanding but also aligns emotions with political ideologies, subtly guiding individuals toward specific viewpoints and shaping perceptions and beliefs. Wilson underscores the role of metaphors in shaping political perspectives, which directly influence how we reason and make decisions (Wilson, 1990). Thus, metaphors are not only essential cognitive tools but also key drivers in political decision-making and ideological persuasion. In this context, metaphors become an essential element of the cognitive structure, contributing to decision-making processes in ways that extend beyond mere linguistic expressions (Gibbs, 2011).

Traditional methods for identifying metaphors, such as corpus-based tools (e.g., Wmatrix and Antconc) and manual annotation, often face challenges like limitations in context, cross-domain mapping, and semantic reasoning. These issues require significant human intervention. To overcome these limitations, it is crucial to explore more advanced and efficient methods of metaphor identification. This paper addresses these challenges by proposing the use of LLMs (Large Language Models) for metaphor identification. Leveraging the advanced text comprehension and generation capabilities of LLMs, we can offer a more efficient and nuanced approach to analyzing metaphors in political discourse. This study, therefore, contributes to the field by advancing the application of LLMs in the broader sciences, particularly in automating political language analysis. Additionally, it deepens our understanding of metaphors in political discourse, especially in the language of politically charged figures, such as Donald Trump.

In all, this research highlights the crucial intersection of cognitive psychology and conceptual metaphors, emphasizing how metaphors shape not only cognition but also our understanding of political language, ultimately contributing to the broader psychological and cognitive frameworks that guide human thought and behavior.

## 2 Literature review

### 2.1 Metaphor research in embodied cognition

Metaphor research from the perspective of embodied cognition has made significant strides in recent years, with scholars investigating the intricate relationship between embodied cognition and metaphor from various angles. Slepian and Ambady (2014) conducted experimental studies revealing that novel embodied metaphors can trigger embodied simulation, which illustrates the connection between abstract concepts and sensorimotor processes. Similarly, Wang et al. (2024) explored the comprehension of Chinese action-verb metaphors, confirming the embodied effect by demonstrating that reaction times are shorter when action primes align with the metaphoric action-verb. Kim (2013) suggested that conceptual metaphors grounded in embodied cognition provide an alternative to traditional interface limitations, emphasizing the potential of embodied cognition to explain complex interfaces. Additionally, Winter and Yoshimi (2020) examined the philosophical implications of embodied mathematics metaphors, underscoring that abstract mathematical concepts are rooted in tangible physical representations. However, while valuable insights have been gained, existing research still faces limitations, such as the boundaries of embodied cognition, the embodied foundation of abstract concepts, and the influence of culture and task-specific factors on metaphor usage. The application of large language models in metaphor identification offers a promising avenue for advancing metaphor research within the embodied cognition framework, paving the way for further exploration and refinement.

### 2.2 Metaphor identification in cognitive linguistics

The identification of metaphors has shifted from intuition-based traditional methods to more advanced computational approaches that integrate neural networks (Habernal and Gurevych, 2017) and NLP technologies (Zhao and Zhao, 2024). Traditional methods, which depend on subjective judgment, have faced criticism for their lack of objectivity. In contrast, computational methods offer greater precision but are often hindered by high technical barriers, limiting their accessibility. In this context, the Metaphor Identification Procedure (MIP) developed by the Pragglejaz Group became widely used due to its simplicity and ease of implementation (Group, 2007). However, the MIP faces challenges in defining lexical units and establishing consistent criteria for metaphor identification. To address these issues, Steen et al. introduced the MIPVU procedure, which refines the identification process by providing a more detailed segmentation of lexical units and extending the scope to include indirect, direct, and implicit metaphors (Steen et al., 2010). Although the MIPVU method has contributed to a deeper analysis of metaphors, it adds complexity to the identification process and still carries some degree of subjectivity, limiting its widespread use in practical research. The development of automatic semantic analysis tools, such as Wmatrix (Rayson, 2008), has led some scholars to combine Wmatrix with MIP or MIPVU techniques to reduce the subjectivity of manual metaphor identification.

### 2.3 Political metaphor research

Conceptual Metaphor Theory (CMT) suggests that metaphors are intuitively understood because they are deeply rooted in our bodily experiences. Fairclough (2001) argues that the primary aim of Critical Discourse Analysis (CDA) is to investigate how language functions in the creation, perpetuation, and transformation of social power, often overlooking the universal nature of language and its influence on individual consciousness. The integration of CMT with CDA, a field within applied linguistics that explores the intersection of language, ideology, and power, has given rise to the field of “political metaphor studies” (Twardzisz, 2013). In CDA, metaphors hold particular significance due to the understanding that “every metaphor carries with it a particular ideological content” (Fairclough, 2001), making them a crucial tool for revealing the underlying conceptual and ideological structures that CDA aims to uncover.

Aligned with CDA, CMA examines how political leaders, journalists, and public figures unconsciously use and internalize metaphors (Jonathan, 2004), exposing the ideological biases and manipulative effects embedded in their discourse. CMA provides a unique perspective on analyzing the “successful political rhetoric of leaders” (Jonathan, 2004), offering valuable insights into how metaphors shape political discourse and influence public perception.

In the digital era, advances in computational linguistics, NLP, and artificial intelligence (AI)—particularly the breakthroughs in LLMs—have significantly enhanced the efficiency and accuracy of metaphor identification. By leveraging carefully crafted prompts, LLMs can now perform metaphor detection with impressive precision (Cheng, 2023). Compared to traditional metaphor recognition methods based on manual or corpus-based approaches, ChatGPT has several advantages: ChatGPT is pre-trained on large-scale text data, allowing its metaphor recognition to overcome personal subjectivity, making the results more objective and reproducible; ChatGPT processes data at a faster speed, saving substantial labor costs; ChatGPT is continuously updated and iterated, enabling it to adapt to emerging linguistic phenomena and metaphorical expressions (Yu, 2025); ChatGPT is simpler and more convenient to operate, as the prompt engineering can be fine-tuned to suit different corpora, making it suitable for a broader range of language learners, while traditional methods are more complex and heavily rely on the experience and expertise of linguistics specialists.

Therefore, given the various limitations and shortcomings of traditional methods, and the theoretical potential of large language models to overcome these factors, the study will use Trump’s speeches as case studies for empirical research. By comparing results obtained through different methods, the study will profoundly reveal the effectiveness and shortcomings of implementing large language models for automated identification of metaphors, offering insights for further optimization of metaphor recognition methods.

## 3 Data sources and method

### 3.1 Data

The study selects four speeches delivered by Donald Trump before and after his campaign for the 47th presidency of the United States as the corpus. These include: the speech on July 18, 2024, in Wisconsin, where he accepted the Republican nomination (notably, this speech was given just 1 week after he was shot); the speech on November 6, 2024, following his victory in the U. S. presidential election; the inaugural speech on January 20, 2025, following his swearing-in as President of the United States; and the speech on March 5, 2025, during a joint session of Congress. Spanning over a year of Trump’s presidential campaign, these speeches cover a variety of occasions and thematic content, offering a comprehensive portrayal of his rhetorical style. They contain numerous vivid, flexible, and highly inflammatory political metaphors, providing ample primary material for this study. The total word count of the corpus is 28,127 words, with 127,932 characters. Throughout the speech, Trump strategically employs a variety of linguistic and rhetorical devices, including metaphors, analogies, and exaggerations, to underscore his political agenda and effectively communicate his message. Given the rich diversity and complexity of the metaphors within Trump’s rhetoric, this dataset offers an ideal foundation for analyzing political metaphors through large language models. It provides an invaluable opportunity for researchers to investigate how political discourse—especially metaphorical language—is systematically deployed to shape public perception and influence political behavior.

### 3.2 Methodology

#### 3.2.1 CMA

The central tenet of CMA is that metaphors in discourse serve not only as rhetorical devices but also as critical instruments for articulating ideology and rhetorical motivations. CMA involves a rigorous examination of questions such as: “Why is this a metaphor? What type of metaphor is it? What motivates the choice of this metaphor?” This analytical framework addresses the criteria for metaphor identification, typological features, intended functions, and the role of metaphors within discourse. By focusing on the communicative goals and contextual factors surrounding the speaker, CMA seeks to uncover the underlying motivations for metaphor selection. Ultimately, this approach provides valuable insights into the ideologies, attitudes, and beliefs that inform and shape the discourse.

#### 3.2.2 PE based on chain of thought (CoT)

In human-AI interaction, the prompt serves as the initiating point for engaging with LLMs, thereby directly influencing the scope and depth of tasks such as metaphor identification. As such, the construction of precise and well-organized prompts is crucial for optimizing the quality of recognition outcomes. PE is a technique that involves providing specific instructions or cues to pretrained language models to steer them towards generating the desired output (Floridi and Chiriatti, 2020). Notably, CoT-based prompt engineering has emerged as a novel methodology for enhancing the performance of large language models (Wei et al., 2022). By incorporating systematic reasoning processes, CoT-based prompts promote greater analytical transparency, logical coherence, and depth. While traditional approaches often yield direct answers to complex inquiries, CoT techniques require the model to articulate its reasoning and rationale at each step, thereby enhancing the transparency and interpretability of the research process. In the context of metaphor analysis, for example, researchers can design multi-step prompts that guide the model through identifying, interpreting, explaining, and analyzing metaphors in relation to their social and cultural contexts, ultimately revealing deeper layers of meaning.

### 3.3 Framework

**Figure 1:**
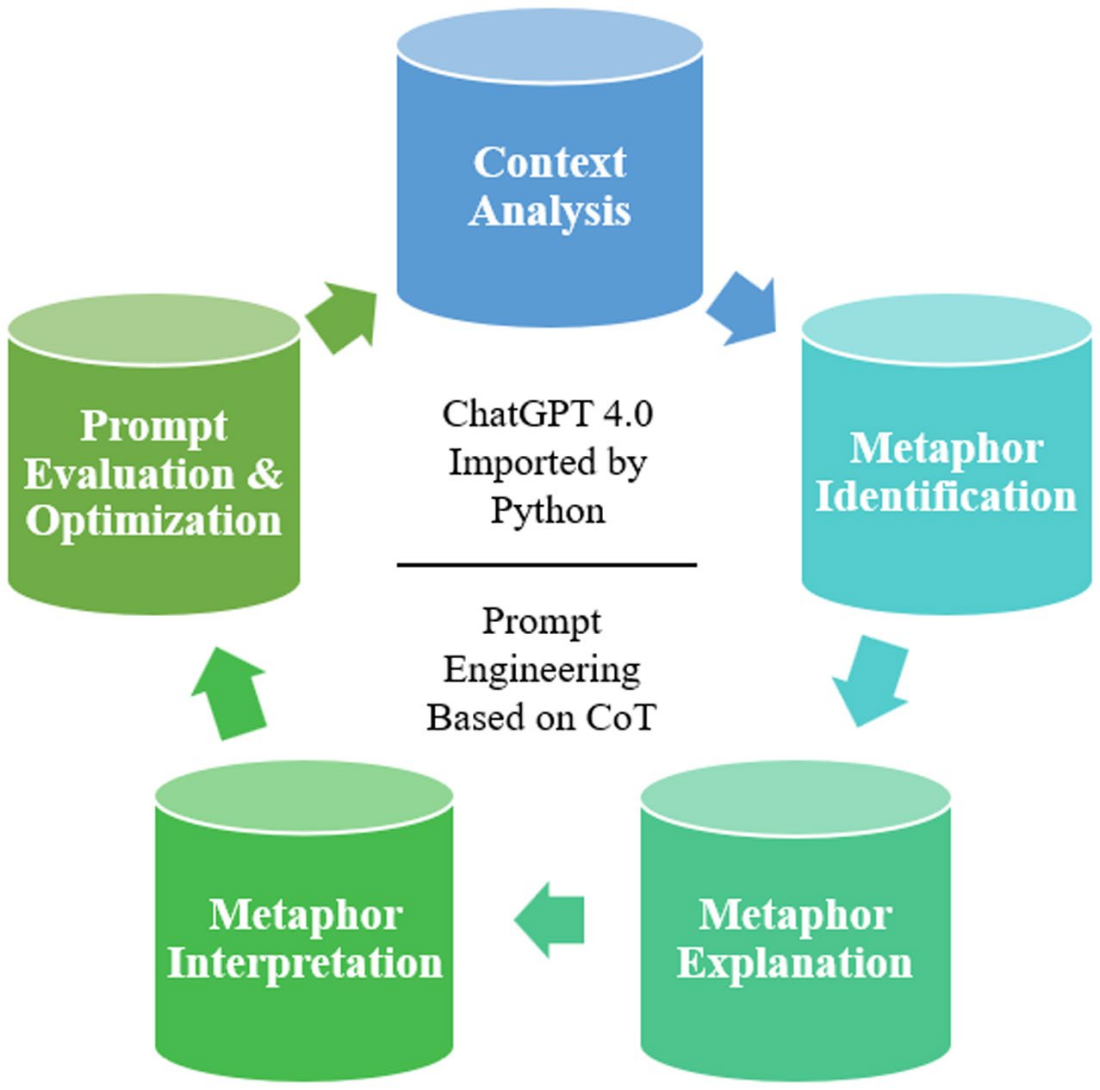
Research flowchart.

This study aims to leverage the CMA framework to identify and analyze metaphors in Donald Trump’s political speeches; while exploring the ideological, socio-cultural, and potential effects these metaphors may have on audience attitudes. The research not only focuses on the identification of metaphors but also examines how these metaphors reflect political viewpoints, social conflicts, and shape public sentiment. To achieve this, we have designed a Prompt-based framework grounded in CMA theory, with the objective of automating the identification and analysis of metaphors in text using OpenAI’s ChatGPT-4.0. The framework follows the four core steps of CMA—contextual analysis, metaphor identification, metaphor explanation, and metaphor interpretation—while integrating CoT techniques to guide the model through a step-by-step reasoning process. Figure 1 depicts the design of the framework for this study. To maintain consistency in output format and facilitate subsequent processing, all steps will be implemented using Python: the source text is first segmented into 1,000 (about)-word chunks using textwrap and os modules, then sequentially fed into ChatGPT API via requests library. Each API response is programmatically aggregated into a unified TXT file through iterative batch processing, while final analysis results are structured in TXT or formats for systematic handling. This framework facilitates not only a comprehensive examination of metaphors in Trump’s speeches but also illuminates the role of metaphors within political discourse. Consequently, it offers both theoretical insights and methodological support for the analysis of metaphors and the broader understanding of language.

#### 3.3.1 Context analysis

**Figure 2:**
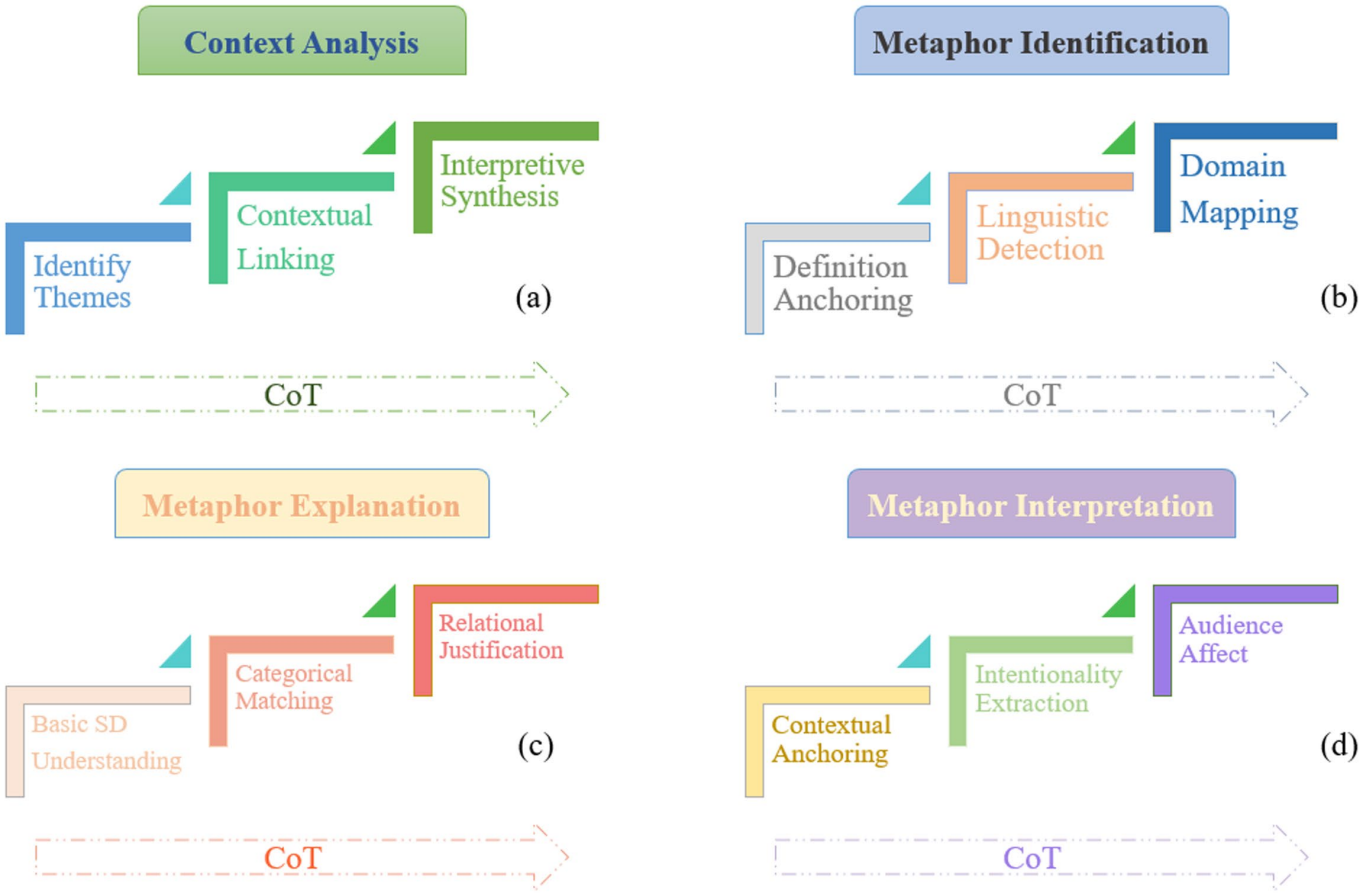
CoT of critical metaphor analysis.

At this stage, the primary objective of the prompt is to guide the model in comprehending the broader context of the text, with particular emphasis on identifying the political, social, and cultural dimensions of the speech. This approach first directs the model to identify core textual themes, then progressively links these themes to contextual factors (historical precedents, social conflicts), and finally evaluates how such contextualization shapes metaphorical interpretations (Figure 2a). The model is expected to discern the central themes of the text, examine the relevant contextual factors, and identify any underlying ideological frameworks. A representative example of the prompt is as follows: “Based on the following text, identify the potential social and historical backgrounds. Begin by outlining the core themes of the text, then analyze the relationship between these themes and relevant historical events, and finally, discuss how these contextual factors may shape the use and interpretation of metaphors.”

#### 3.3.2 Metaphor identification

Following the contextual analysis, the model’s pre-existing knowledge is activated to identify metaphors within the text through a CoT framework that hierarchically structures metaphor detection: conceptual grounding in metaphor theory; linguistic pattern recognition; and domain mapping validation. Thus, the prompt is designed to guide the model through a three-stage cognitive sequence (as seen in Figure 2b). This includes defining what constitutes a metaphor and clarifying the source and target domains within the metaphorical expressions. A representative example of the prompt is as follows: “First, provide a clear definition of metaphor and specify which sentences can be considered metaphorical. Then, identify the phrases or sentences in the following text that are metaphorical in nature. Finally, explain why these expressions qualify as metaphors and identify their respective source and target domains.”

#### 3.3.3 Metaphor explanation

In this stage, the identified metaphors are categorized and mapped to the relevant source domains through a CoT trajectory that decomposes domain mapping into sequential reasoning layers: first internalizing theoretical taxonomy initiated by Kovecses (2010), then validating category alignment, and finally establishing cross-domain semantic coherence (Figure 2c). Drawing on the 13 common source domains proposed by Kövecses (The Human Body, Health and Illness, Animals, Plants, Buildings and Construction, Machines and Tools, Games and Sport, Money and Economic Transactions, Cooking and Food, Heat and Cold, Light and Darkness, Force, Movement and Direction), the prompt designed follows this logic: “The 13 common source domains are as outlined below. Begin by comprehensively understanding and explaining the essential meanings of these source domains. Then, for each identified metaphor, map it to the corresponding source domain, providing a rationale for why the target domain is appropriately linked to the chosen source domain.”

#### 3.3.4 Metaphor interpretation

Following the interpretation of metaphors, we further investigate their role within socio-cultural contexts, focusing on how they shape public emotions, attitudes, and cognition, which is structured to guide a tripartite analytical trajectory—contextual anchoring (linking metaphors to social hierarchies) → intentionality extraction (decoding authorial purpose) → audience affect mapping (see Figure 2d). The prompt is designed as follows: “Please explain how metaphors operate within a socio-cultural context. Begin by analyzing the relationship between this particular metaphor and social class, political power, or cultural values. Next, deduce the emotions and ideas the author seeks to convey through the metaphor. Finally, elucidate how this metaphor may influence the emotions and cognitive responses of the audience.”

#### 3.3.5 Prompt evaluation and optimization

After the initial analysis, the prompts should undergo evaluation and refinement. Preliminary results may reveal inaccuracies or insufficient depth, necessitating adjustments to the prompt design to enhance the precision of metaphor identification and improve the overall depth of the analysis.

## 4 Results and discussion

### 4.1 Macro understanding

An analysis of former President Donald Trump’s four speeches, conducted with the aid of Python and ChatGPT-4.0, offers valuable insights into his strategic use of metaphors within both historical and social contexts.

#### 4.1.1 Theme and background identification

The speeches are underpinned by several key themes, including election victory, social unity, immigration policy, economic strategy, law and order, international relations, political criticism, and personal experience. These themes are essential for understanding the metaphorical impact of the speeches, as each one elicits distinct emotional responses that shape the audience’s perception.

#### 4.1.2 Metaphor usage and its influence

Metaphors throughout the speeches play a pivotal role in shaping the audience’s understanding of key political issues. Those linked to election victory and the nation’s future invoke feelings of optimism and pride, and some addressing social division and unity emphasize the need for collective action and healing. Immigration-related metaphors focus on security and economic stability, while those addressing economic issues, particularly energy policy, align with themes of self-sufficiency and growth. Law-and-order metaphors highlight the importance of public safety, and metaphors of international conflict underscore assertive leadership. Political criticism, framed through metaphors of incompetence, bolsters Trump’s image as a capable leader. Finally, metaphors drawn from personal experience and faith emphasize resilience and divine guidance, resonating deeply with the audience’s values.

Together, these metaphorical themes construct a powerful narrative that not only appeals emotionally but also aligns ideologically with the audience, reinforcing Trump’s political platform and rallying support for his campaign.

### 4.2 Refined identification

**Figure 3:**
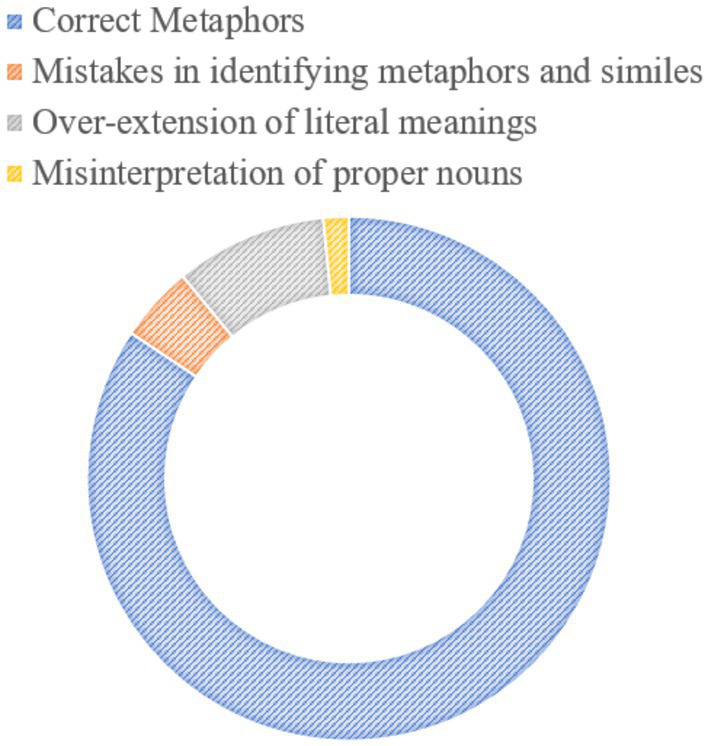
Pie chart for metaphor identification.

Following the processing of the text file exported by the Python script, a total of 138 sentences were analyzed. A thorough evaluation by human experts identified 119 sentences as containing metaphorical expressions, while the remaining 19 were classified as non-metaphorical (see Figure 3).

To assess the precision of CoT-based prompt engineering in metaphor recognition tasks, we applied the following formula:


$$\left[\text{Accuracy}=(\begin{array}{l} \text{Number of metaphorical sentences}/\\ \text{Total number of sentences} \end{array})\times 100\%\right]$$

This calculation yielded an accuracy rate of 86.2%, indicating that prompt engineering demonstrates a reasonable level of precision and reliability in metaphor identification. However, this result also suggests that there is room for further optimization.

Upon examining the misclassification of non-metaphorical sentences, three primary factors were identified as contributing to errors:

- Blurring the Distinction Between Metaphor and Simile. LLMs often encounter difficulties in distinguishing metaphors from similes, particularly in sentences containing the verb “to be.” For instance, in the sentence, “Washington D. C., which is a horrible killing field,” the model may incorrectly interpret the verb “is” as signaling a metaphor, failing to recognize its function in a literal statement. In reality, “killing field” serves as a direct simile, describing a violent, chaotic environment.
- Overextension of Literal Meanings. The model occasionally extends the literal meanings of words beyond their intended scope, misclassifying everyday expressions as metaphors. For example, in the sentence “It’s a series of bold promises that we will swiftly implement,” the term “series” does not carry metaphorical connotations, but the model might erroneously interpret it as a metaphorical reference to “spatial linkage” or an abstract comparison.
- Misinterpretation of Proper Nouns. The model may struggle to differentiate between proper nouns and metaphorical expressions due to a lack of sufficient contextual or domain-specific knowledge. For example, in the case of “Iron Dome,” a missile defense system, the model might mistakenly categorize the term as metaphorical, failing to recognize it as a reference to a specific technological system rather than a symbolic expression.

In conclusion, while the model demonstrates promising accuracy in metaphor detection, these sources of misclassification highlight key areas for refinement in order to enhance its overall performance.

### 4.3 Deep classification

**Figure 4:**
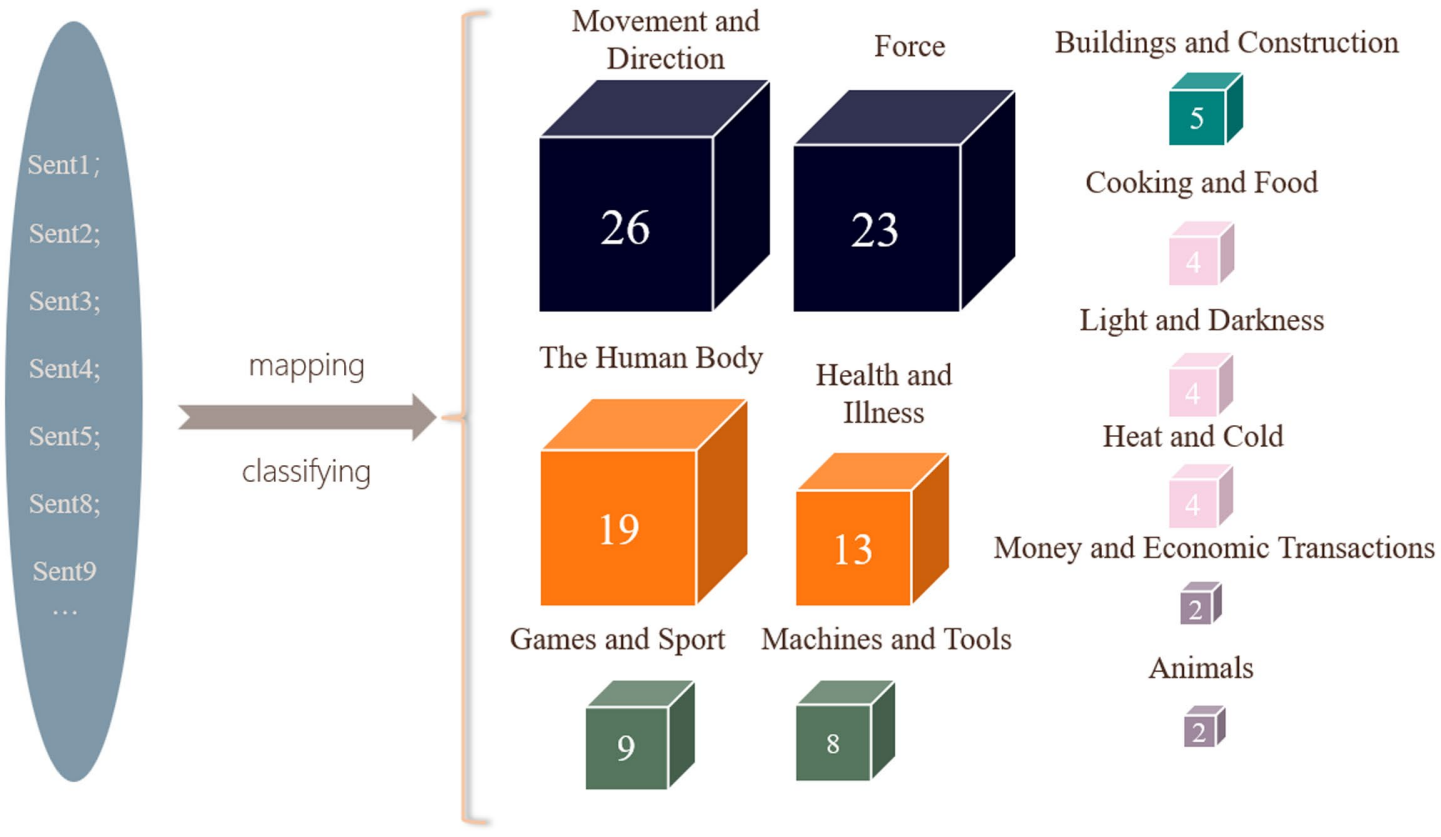
Metaphor classification.

Grounded in metaphor identification, the analysis employs Kövecses’s framework of 13 common conceptual domains as a basis for categorizing metaphors. The research primarily explores how fine-tuned prompts can effectively identify metaphors within the speech and systematically map these metaphors to their corresponding source domains, generating structured JSON data, which is subsequently visualized (see Figure 4).

Each source domain corresponds to a category of metaphors sharing similar characteristics, offering insights into the deep relationship between language and cognition. For instance, the Movement and Direction domain employs kinetic verbs like “Americans pushed thousands of miles through wilderness” “Our spirit is back” “We rise together or we fall apart” to recast policy agendas as frontier migrations, positioning social reforms as returns to mythologized origins through phrases like “bring law and order back.” These metaphors underscore the significance of national progress or decline and the collective effort required, resonating emotionally with the audience regarding the nation’s future trajectory. Another prominent domain, the Force domain, manifests through visceral imagery such as “Unlock America’s glorious destiny” and “A tide of change is sweeping the country,” where legislative actions are framed as geological forces reshaping national terrain. The declaration “We have taken back control of the Senate” spatializes political power into conquerable territory, while “sunlight pouring over the world” transforms ideological expansion into solar inevitability. These metaphors not only convey the exercise of political power but also reinforce the connection between national security, prosperity, and freedom, galvanizing audience support for future societal transformation.

The findings reveal that Movement and Direction and Force are the most salient source domains in Trump’s speech, with each containing 26 and 23 metaphorical expressions, respectively. These domains reflect a significant emphasis on the nation’s future direction, political power, and social change. In contrast, the Human Body and Health and Illness domains are also notably represented, with 19 and 13 metaphorical expressions, respectively. Biological analogies permeate the discourse, with The Human Body metaphors such as “Ambition is the lifeblood” and “the spirit of the frontier is written into our hearts” anatomizing national identity, while Health and Illness frames like “help our country heal” and “thriving in hearts” medicalize political conflict. The metaphor “tariffs protect the soul of our country” exemplifies this corporeal logic, equating economic measures with cellular defense mechanisms. Concurrently, thermodynamic imagery bifurcates economic rhetoric—“economy boom” symbolizes productive combustion versus “burning like hell” depicting the risk of uncontrolled threat—demonstrating how temperature metaphors polarize policy outcomes.

Ultimately, the political metaphors transcend rhetorical ornamentation, functioning as cognitive architecture that transforms abstract governance into visceral experiences. By employing metaphors, Trump is able to transform abstract and complex political issues into more tangible concepts, enabling audience to form an intuitive understanding and emotional connection. This linguistic strategy proves particularly effective when addressing sensitive topics such as national destiny, social change, and partisan conflict. Through this process of concretization, metaphors enhance emotional resonance, provoke powerful emotional responses, and significantly bolster the persuasive impact and political appeal of the speech.

### 4.4 Socio-cultural analysis

Metaphors play an important role in shaping political and cultural discourse, influencing public agendas and deeply affecting the emotions, attitudes, and cognition of audiences. This analysis explores the relationship between metaphor categories and societal classes, political power, and cultural values, examining how metaphors convey emotions and ideas and their impact on audience responses.

Metaphors across various domains depict national and societal conditions, evoking emotional resonance. Movement and direction metaphors, such as “rise” and “decline,” symbolize national fate and leadership responsibility, promoting unity and change. Force metaphors, often drawing from war or invasion, create an “us versus them” dynamic, heightening national security concerns. Body metaphors liken the nation to a living organism, emphasizing economic challenges and the need for “healing” solutions. Health and disease metaphors reflect societal contradictions, driving political and social reform. Machine and tool metaphors emphasize resilience, symbolizing recovery and confidence. Sports metaphors frame political struggles as winnable contests, while building metaphors focus on economic reconstruction, symbolizing the protection of national foundations. Food and cooking metaphors highlight economic pressures and resource depletion. Currency metaphors, like “liquid gold,” underscore national resource advantages, fostering economic pride. Light and darkness metaphors depict a nation’s journey from crisis to hope, stimulating optimism for change. Animal metaphors strengthen national identity, while heat and cold metaphors, using “flame” for freedom, emphasize the responsibility to protect liberty.

How Metaphors Convey Emotions, Ideas, and Influence the Audience. These metaphors function in several key areas:

- Eliciting Emotions and Mobilizing Action: Metaphors engage emotions such as fear, hope, and pride, influencing attitudes and behaviors. Movement metaphors, depicting national “rise” or “fall,” encourage collective action and political participation.
- Strengthening Leadership Authority and Legitimacy: Metaphors personify political and social challenges as war, disease, or competition, reinforcing leadership authority and legitimizing policies. This portrayal of leaders as “saviors” builds public trust and motivates support for leadership decisions.
- Influencing Perceptions of Division and Unity: Some metaphors, like disease and invasion, highlight national conflict, evoking dissatisfaction while calling for unity. Conversely, metaphors emphasizing unity and collective effort, such as “rise” or “build,” inspire hope and motivate the public to work toward a better future.

Through these mechanisms, metaphors not only frame complex political and social realities but also function as powerful tools for emotional engagement and cognitive transformation, shaping both individual perceptions and collective action.

### 4.5 Reflection

To evaluate the effectiveness of the CoT-based prompt engineering output, this study combines the corpus tool Wmatrix 5.0 and the MIPVU method for metaphor identification and classification. The results obtained from both approaches are then compared and analyzed to provide recommendations for improving the experiment. The metaphor identification results are presented in Supplementary Tables 1, 2.

**Figure 5:**
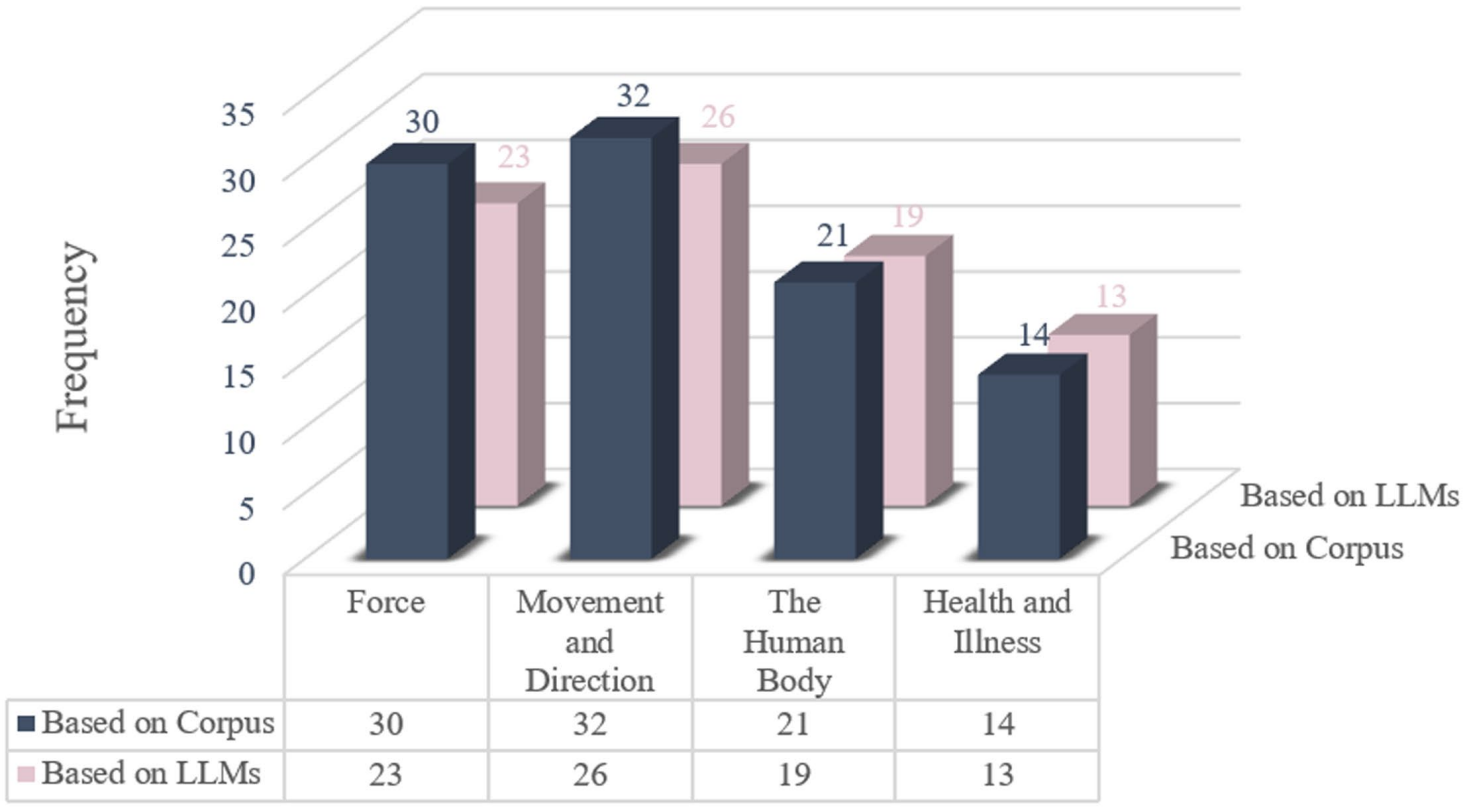
Metaphor recognition results based on different technical approaches.

Based on the two processing modes, metaphor categories with higher frequencies of occurrence were selected for comparative analysis, as presented in Figure 5.

Using the Pearson correlation coefficient formula:


$$\gamma =\frac{n(\mathrm{\Sigma xy})-(\mathrm{\Sigma x})(\mathrm{\Sigma y})}{\surd [\mathrm{n\Sigma}x^{2}-{\left(\mathrm{\Sigma x}\right)}^{2}][\mathrm{n\Sigma}y^{2}-{\left(\mathrm{\Sigma y}\right)}^{2}]}$$

Where, n is the number of data points, x and y represent the two datasets, x = array ([21, 14, 3, 1, 8, 10, 11, 3, 6, 5, 30, 32, 1]), y = array ([19, 13, 2, 0, 5, 8, 9, 2, 4, 4, 23, 26, 4]) and Σ denotes summation. The substitution of the given data gets a value of 0.987, which indicates a very strong positive correlation between the two datasets, suggesting a highly consistent and closely aligned trend. The comparative analysis yields the following insights:

Additionally, we hypothesize that the classification results from the Corpus-assisted Method serve as a reference standard, and calculate the precision, recall, and F1 scores for the ChatGPT-assisted Method across each category. The following analysis is based on this assumption (with the number of categories in the Corpus corresponding to the actual sample sizes and the number of classifications by ChatGPT representing the predicted values).

#### 4.5.1 Confusion matrix construction assumptions

For each category *i*:

- True Positives (TP): The number of instances correctly classified into category *i* by ChatGPT (taken as the smaller of the two values).
- False Positives (FP): The number of instances incorrectly classified into category *i* by ChatGPT (if the number predicted by ChatGPT exceeds that of the Corpus, the difference is counted as FP).
- False Negatives (FN): The number of instances from category *i* that were missed by ChatGPT (if the number in the Corpus exceeds that of ChatGPT, the difference is counted as FN).

The result table is presented in Supplementary Table 3.

#### 4.5.2 Analysis results

##### 4.5.2.1 Overall consistency

In most categories, ChatGPT demonstrated high precision, with perfect accuracy in areas like The Human Body and Health and Illness (1.00), indicating reliable predictions and no misclassifications. However, recall varied significantly across categories. For instance, Health and Illness had a recall rate of 0.9286, showing that ChatGPT correctly identified most true samples. In contrast, categories like Plants had a recall rate of 0.00, suggesting that ChatGPT failed to recognize any relevant samples. When it comes to metaphor recognition, the model showed considerable accuracy, especially in categories such as Force, Movement and Direction, and The Human Body and Health and Illness. LLMs not only independently identify metaphors but also complement existing NLP recognition methods by cross-validating predictions.

##### 4.5.2.2 Typical category analysis

The Cooking and Food category yielded some unexpected results. ChatGPT predicted 4 samples, but only 1 aligned with the Corpus, leading to a high false positive rate (3). The precision for this category was low (0.25). However, with just 1 sample in the Corpus, the recall rate was perfect (1.00), indicating that ChatGPT correctly identified the one true sample. This highlights the challenges LLMs face when dealing with small or ambiguous sample sizes, as seen in metaphor detection tasks with unclear references. The Plants category posed a more significant problem, with no predictions made (predictions = 0), resulting in a recall rate of 0. This illustrates the difficulties LLMs encounter when identifying metaphors with ambiguous or vague references, much like how phrases in Donald Trump’s speeches, such as “I brought taxes way down, way, way down,” were incorrectly labeled as metaphors in the Movement and Direction category. These challenges emphasize the need for human annotation to improve accuracy.

#### 4.5.3 F1 score distribution

Most categories showed F1 scores above 0.8, demonstrating ChatGPT’s strong performance in balancing precision and recall. Categories like The Human Body (0.950) and Health and Illness (0.963) stood out with excellent results. However, in more specialized or challenging categories, such as Plants (0.00) and Cooking and Food (0.400), the F1 scores were much lower, highlighting areas where ChatGPT struggles. Likewise, while LLMs perform well in metaphor detection, they face difficulties in identifying more subtle or complex metaphors. The combined approach of MIPVU and Wmatrix 5.0 proves to be broadly applicable, but human involvement is still crucial to ensure accuracy, particularly in more intricate metaphor detection scenarios.

### 4.6 Limitations and optimizations

Although large language models have demonstrated their strong potential in handling more complex metaphor recognition tasks, however, as of now, there are still several issues to be solved.

Firstly, the datasets used for pre-training existing LLMs are primarily composed of publicly scraped, unannotated raw corpora, which vary greatly in quality. Additionally, the corpus provided by users during human-computer interaction is relatively limited in scale (Xu et al., 2025), leading to frequent obstacles in accurately identifying the metaphor types in target texts. Although pre-training LLMs with cleaned and annotated text data can make them more sensitive to specific metaphorical patterns, the existing metaphor datasets are often limited by particular domains or contexts. This can result in poor generalization in cross-domain or multilingual environments. Furthermore, the expression of metaphors varies across different cultural, social, and linguistic contexts, and current datasets struggle to comprehensively cover all metaphorical variants.

Secondly, LLMs often exhibit stereotypes or biases (De Vassimom Manela et al., 2021), such as language bias, demographic bias, and evaluative bias, which can be transferred into the downstream tasks of LLMs (Xu et al., 2024), shaping their linguistic performance during human-computer interactions. As a result, in the four-step workflow of metaphor identification, LLMs may project their biases related to gender, race, occupation, culture, and other factors onto some or all parts of the process, making it difficult to accurately identify metaphorical expressions from certain fields or groups.

Additionally, current mainstream LLMs, such as ChatGPT-4, exhibit high prompt sensitivity, meaning that even small changes in the prompts can lead to significantly different outputs. Metaphor identification is a complex linguistic task, and its effectiveness is significantly influenced by how the prompts are constructed. Different metaphor types may require distinct prompting strategies to guide the model to analyze the metaphors from specific perspectives, making the model’s application unstable and unpredictable.

To specifically address the three issues listed above and improve LLM-based metaphor recognition, the following strategies are proposed:

To address the issues of cross-domain and cross-cultural adaptability faced by LLMs in metaphor recognition, it is necessary to expand and diversify the training datasets, especially high-quality datasets that have been cleaned and annotated, for pretraining the LLMs. This will enable the model to learn a broader range of metaphorical expressions, thereby improving its ability to recognize metaphors in different contexts and cultural backgrounds. While expanding the dataset, it is also essential to ensure the balance of the dataset. The number of metaphors in various metaphor types covered by the dataset should be relatively balanced, which can effectively avoid overfitting of certain metaphor types and improve the model’s accuracy in recognizing all metaphor types.

Regarding the issue of bias in large language models, bias-reduction techniques can be used to mitigate the model’s bias towards specific groups based on gender, race, occupation, etc., during training. An effective approach is to preprocess the dataset with bias-reduction techniques, such as counterfactual data augmentation, which involves creating texts that contradict existing facts to supplement the training data, thereby alleviating bias caused by class imbalances. Simultaneously, establishing a diversified validation and review mechanism is crucial. By introducing cross-cultural and cross-lingual validation teams, a comprehensive and multi-dimensional review of the metaphor recognition results can be conducted to ensure that the model performs fairly and accurately across various cultural contexts.

To address the issue of prompt sensitivity in large language models, the use of standardized prompt templates can help reduce fluctuations in recognition results caused by changes in prompts. Through systematic adjustments and experimentation with different types of prompt designs, the most suitable approach to guide the model in metaphor recognition can be identified. Additionally, to further improve the model’s stability and accuracy, an adaptive prompt adjustment mechanism can be developed, allowing the model to automatically adjust the content and structure of prompts based on the different characteristics of the text and metaphor types. This approach ensures that the model can make reasonable analyses based on the actual context, whether the metaphor is a complex political metaphor or a more symbolic literary metaphor, thereby enhancing both the stability and accuracy of recognition.

Adopting these solutions will help improve the model’s generalization ability, reduce bias, enhance stability, and further advance the application of large language models in metaphor recognition, promoting their development and progress in broader fields of linguistics and social science.

## 5 Conclusion

A framework to analyze metaphor usage in Donald Trump’s four speeches, highlighting the pivotal role of metaphors in political discourse. The findings reveal how Trump’s metaphorical strategies effectively evoke emotional resonance, using source domains like Movement and Direction and Force to emphasize national progress, political strength, and social transformation, thereby enhancing the persuasive impact of his speeches.

Methodologically, while LLMs offer significant strengths in analyzing political contexts and the socio-cultural dimensions of metaphors, challenges remain in distinguishing metaphors from similes, ensuring consistency, and addressing complex referential contexts. Future research should compare LLMs-based approaches with traditional metaphor identification methods, refine model accuracy, and explore their applicability across diverse political texts and cultural settings. Incorporating sentiment analysis could also deepen our understanding of how metaphors influence audience emotions and cognition.

Longitudinal studies are needed to track the evolution of political metaphors and assess their long-term impact on discourse and societal attitudes. This research not only sheds light on Trump’s rhetorical strategies but also opens avenues for further applications of LLMs in broader science research, with the aim of refining metaphor analysis techniques and enhancing the study of cognitive linguistics.

## Data Availability

The original contributions presented in the study are included in the article/Supplementary material, further inquiries can be directed to the corresponding author.
