# Supplementary material for: Large language models prompt engineering as a method for embodied cognitive linguistic representation: a case study of political metaphors in Trump’s discourse
Source: Front Psychol. 2025 Jun 25;16:1591408. doi: 10.3389/fpsyg.2025.1591408 (PMC12239739; doi:10.3389/fpsyg.2025.1591408)
Supplement: Supplementary file 1 [file Table_1.docx]

Supplementary Material

Large Language Models Prompt Engineering as a Method for Embodied Cognitive Linguistic Representation: A Case Study of Political Metaphors in Trump’s Discourse

Haohan Meng^1†^, Xiaoyu Li^1†^, Jinhua Sun^*^

*** Correspondence:** Jinhua Sun: tracy758@163.com

## Supplementary Table

| Source Domain | Metaphorical Bearer and Frequency |
| --- | --- |
| The Human Body (21) | blood (4), heart (3), hand (2), head (2), soul (2), backbone (1), fist (1), ear (1), eyes (1), legs (1), wounds (1), brain (1), spine (1), breath (1), voice (1), fists (1), woke (1), body (1), arms (1), guts (1), nerves (1) |
| Health and Illness (14) | heal (3), disease (2), cancer (2), virus (1), cure (1), pandemic (1), immunity (1), vaccine (1), poison (1), infection (1), symptoms (1), recovery (1), epidemic (1), trauma (1) |
| Animals (3) | roar(2), lambs(1) |
| Plants (1) | trim(1) |
| Buildings and Construction (8) | build (3), foundation (2), demolish (1), structure (1), pillar (1), blueprint (1), fortress (1) |
| Machines and Tools (10) | engine (2), drill (2), weapon (2), tool (1), gear (1), fix (1), unbreakable (1), tank (1), shield (1), lever (1) |
| Games and Sport (11) | victory (3), win (2), fight (2), beat (2), team (1), score (1), play (1), race (1), champion (1) |
| Money and Economic Transactions (3) | debt (1), wealth (1), invest (1), tariff (1) |
| Heat and Cold (6) | fire (3), burn (2), freeze (1), melt (1), heat (1) |
| Light and Darkness (5) | flame (2), darkness (1), nightmare (1), dawn (1), shadow (1) |
| Force (30) | power (5), strength (3), attack (3), destroy (2), crush (2), pressure (2), resistance (1), obliterate (1), flood (1), squeeze (1), fuel (1), unleash (1), sway (1), slow (1), stop (1), fight (4), win (2), beat (2), strike (1), dominate (1), explode (1), defend (1), conquer (1), shield (1), clash (1), collision (1), momentum (1) |
| Movement and Direction (32) | path (5), journey (4), forward (3), back (3), turn (2), rise (2), fall (2), direction (2), cross (2), climb (1), descend (1), obstacle (2), verge (1), cusp (1), destiny (1), path (1), road (1), way (1), flow (1), redirect (1), pour (1), surge (1), drift (1), escape (1), retreat (1), advance (1), detour (1), pivot (1), orbit (1), launch (1) |
| Cooking and food (1) | eat alive (1), recipe (1), feast (1) |
|  | |

**Supplementary Table 1. Results of Metaphor Recognition Based on CORPUS WMATRIX5.0 + MIPVU**

| Source domain | Metaphorical Bearer and Frequency |
| --- | --- |
| The Human Body (19) | heart (3), soul (2), lifeblood (1), written into hearts (2), thrive (1), bled (1), arteries (1), backbone (1), shield (1), pulse (1), immune (1), veins (1), wounds (1), heal (1), limbs (1), breath (1), spirit (2) |
| Health and Illness (13) | heal (4), killer (2), disease (3), epidemic (1), suffering (2), cure (1), diagnose (1), immunity (1), pathologize (1), recover (1), survival (1) |
| Animals (2) | roar (1), stampede (1) |
| Plants (0) |  |
| Buildings and Construction (5) | forge (2), edge (1), foundation (1), pillar (1), architecture (1) |
| Machines and Tools (8) | weaponization (3), engine (2), tank (1), drill (2), forged (1), machinery (1), gears (1) |
| Games and Sport (9) | fight (4), victory (3), arena (1), beat (1), score (1), play (1), champion (1), team (1) |
| Money and Economic Transactions (2) | liquid gold (1), tariff (1) |
| Heat and Cold (4) | flame (2), burning (1), melt (1), freeze (1) |
| Light and Darkness (4) | golden (2), sunlight (1), dawn (1), shadows (1) |
| Force (23) | launch (3), forge (2), weaponizing (4), invasion (3), flood (2), wiped out (2), fueling (2), unleash (2), push (3), tide (2), swept (1), crush (1), obliterate (1), dominate (1), repel (1), annihilate (1), surge (1), strike (1) |
| Movement and Direction (26) | together…apart (2), flying (3), turn around (2), decline (2), lead to (3), soar (2), drive down (2), pouring (2), current (1), pushed (3), bring back (2), swept (1), surged (1), launch (1), restore (1), anchor (1), propel (1), return (1), reclaim (1), journey (1), march (1), trajectory (1), staked (1), raced (1), trekked (1) |
| Cooking and food（4） | feast (1), recipe (1), boil (1), dinner(1) |
|  | |

**Supplementary Table 2. Results of Metaphor Recognition Based on LLMS (GPT4.0)**

| Category | Quantity (G) | TP | FP | FN | Precision (P=TP/G) | Recall (R=TP/C) | F1 Score |
| --- | --- | --- | --- | --- | --- | --- | --- |
| The Human Body | 21 | 19 | 19 | 0 | 2 | 1.00 | 0.950 |
| Health and Illness | 14 | 13 | 13 | 0 | 1 | 1.00 | 0.963 |
| Animals | 3 | 2 | 2 | 0 | 1 | 1.00 | 0.800 |
| Plants | 1 | 0 | 0 | 0 | 1 | 1.00* | 0.00 |
| Buildings and Construction | 8 | 5 | 5 | 0 | 3 | 1.00 | 0.769 |
| Machines and Tools | 10 | 8 | 8 | 0 | 2 | 1.00 | 0.889 |
| Games and Sport | 11 | 9 | 9 | 0 | 2 | 1.00 | 0.900 |
| Money and Economic Transactions | 3 | 2 | 2 | 0 | 1 | 1.00 | 0.800 |
| Heat and Cold | 6 | 4 | 4 | 0 | 2 | 1.00 | 0.800 |
| Light and Darkness | 5 | 4 | 4 | 0 | 1 | 1.00 | 0.889 |
| Force | 30 | 23 | 23 | 0 | 7 | 1.00 | 0.868 |
| Movement and Direction | 32 | 26 | 26 | 0 | 6 | 1.00 | 0.897 |
| Cooking and Food | 1 | 4 | 1 | 3 | 0 | 0.25 | 0.400 |
| Note: When the quantity of ChatGPT is 0 (e.g., in the Plants category), precision is defined as 1 (no false positives), and recall is 0. | | | | | | | |

**Supplementary Table 3. Results of Confusion Matrix**
